# Genetically predicted circulating serum homocysteine levels on osteoporosis: a two-sample mendelian randomization study

**DOI:** 10.1038/s41598-023-35472-2

**Published:** 2023-06-04

**Authors:** ChenYu Wang, Xiang Zhang, Bo Qiu

**Affiliations:** 1 Department of Orthopedics, Renmin Hospital of Wuhan University, Wuhan, China; 2 Department of Geriatrics, Renmin Hospital of Wuhan University, Wuhan, China

**Keywords:** Genetics, Biomarkers, Diseases, Endocrinology

## Abstract

To investigate the causal relationship between circulating serum homocysteine (Hcy) levels and osteoporosis (OP). Using public datasets gathered from independently published genome-wide association studies (GWAS), Mendelian randomization (MR) analysis was done to investigate the causal influence of Hcy on OP. SNPs were selected from a meta-analysis of GWAS on Hcy concentrations in 44,147 individuals of European ancestry. Meanwhile, SNPs of individuals of European descent for OP were extracted from the Genetic Factors of Osteoporosis Consortium (GEFOS) UK Biobank. The odds ratio (OR) of inverse variance weighted (IVW) approaches was established as the primary outcome. Moreover, weighted median (WM) and MR-Egger regressions were included in the sensitivity analysis. There were no causal effects of Hcy on forearm bone mineral density and lumbar bone mineral density according to IVW, MR-Egger, and WM analyses (all *p* > 0.05). In the IVW, we discovered the causality between genetically predicted Hcy and heel bone mineral density (H-BMD) with an OR of 0.96 [95% confidence interval (CI) = 0.927–0.990, *p* = 0.011]. In the additional sensitivity analysis, WM regression (OR = 0.97, 95% CI = 0.995–1.076, *p* = 0.084) and MR-Egger regression (OR = 0.98, 95% CI = 0.918–1.049, *p* = 0.609) yielded values that were comparable in direction but less precise. The MR-Egger intercept, funnel plot, and IVW all indicate the absence of any discernible directional pleiotropy. The leave-one-out analysis revealed that a single SNP did not influence the results of the MR analysis. In conclusion, our MR investigation revealed evidence of a causal relationship between circulating serum Hcy levels and H-BMD, but not OP in the European population. However, larger sample sizes are needed in the future to get more reliable conclusions.

## Introduction

Osteoporosis (OP) is a worldwide health issue defined by bone microstructure degeneration and low bone mineral density (BMD), which may lower bone strength and increase bone brittleness^1^.

Recent studies have demonstrated a positive correlation between age and OP prevalence, with an incidence rate of 3.2% in individuals aged 40–49 and 32.5% in those aged 65 and above, highlighting the considerable risk OP poses to the physical and mental well-being of middle-aged and elderly individuals, substantially impacting their daily functioning^2^. Therefore, early detection and intervention, as well as effective control of the progression of osteoporosis, are essential.

Methionine metabolism results in the formation of a sulfur-containing amino acid, homocysteine (Hcy). It is affected by genetics, age, vitamin B_12_, folic acid, vitamin B_6_, and other factors and is mainly used in clinical risk prediction of cardiovascular and cerebrovascular diseases. Recent research has linked elevated Hcy levels to decreased bone density^3^. The relative risk of fracture increases with increased serum Hcy concentration^4,5^. According to some studies, high Hcy may affect bone matrix and diminish bone quality by inhibiting cross-linking between collagen fibers and hindering the formation of a collagen network structure^6,7^. Mild to moderately elevated Hcy levels have been shown to activate osteoclast formation and function by generating intracellular reactive oxygen species (ROS) in the bone marrow, resulting in increased bone resorption. Additionally, elevated Hcy levels have been linked to decreased osteocalcin expression and increased osteopontin expression, which disrupts the normal function of osteoblasts, resulting in decreased bone formation and ultimately contributing to osteoporosis^8,9^. Numerous studies have postulated that higher serum Hcy levels may be a risk factor for osteoporotic fractures in the elderly. However, the results from these studies have yielded conflicting findings regarding the relationship between Hcy, BMD, and fractures. After controlling for age and gender, a Dutch study of middle-aged and elderly persons over 55 revealed that Hcy was not associated with bone mineral density. After correcting for age, gender, body mass, and other variables, fracture's relative risk (RR) rose by 1.4 when the natural logarithm of Hcy increased by 1 standard deviation, with no significant gender difference^4^. Similar results were found in a 7-year Swedish study of 996 women (75 + years)^5^. High Hcy levels were not significantly associated with fractures, and the highest quartile of Hcy levels had lower femoral neck BMD and greater trochanteric BMD than the other groups and lower lumbar spine BMD (L2–L4)^10^. After adjusting for parathyroid hormone, smoking, and recent fractures, high Hcy levels were not correlated with low femoral neck BMD^10^.

The variability in diagnostic criteria for homocysteinemia and osteoporosis among the studies and the limited number of individuals with hyperhomocysteinemia could be potential reasons for the inconsistent findings reported. Moreover, traditional observational studies can suffer from confounding bias, leading to discrepant results and concerns. Additionally, the temporal sequence between exposure and outcome is often misinterpreted, further complicating results interpretation. To address these issues, Katan proposed Mendelian Randomization (MR) research^11^.

**Figure 1:**
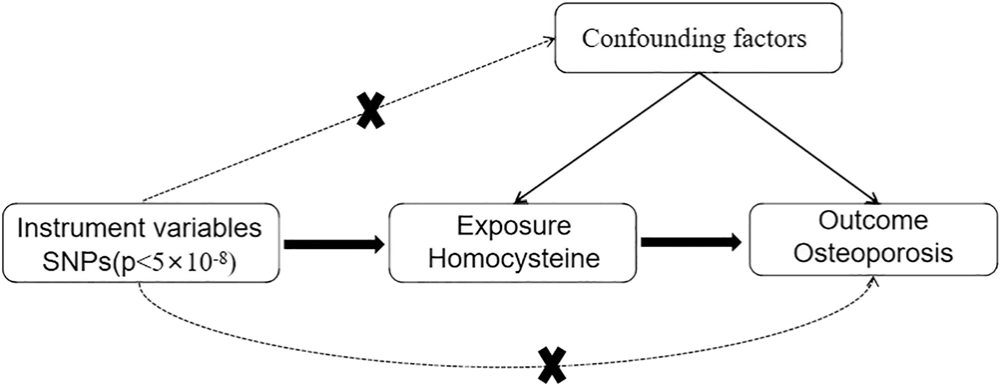
Diagram depicting two-sample Mendelian randomization analysis.

MR is a method that uses instrumental variables (IVs) to examine exposure factors and simulates the influence of exposure factors on diseases by using the causal relationship effect between genotype and disease^12^. Given the random distribution of alleles during gamete formation, the association between genetic variations and diseases is less likely to be influenced by confounding bias and reverse causation compared to traditional epidemiological studies. Therefore, causal inference using genetic data has distinct advantages^13^. As a result, we postulated that this method could uncover causal relationships between Hcy and OP, thus offering further insights into the etiology of OP (Fig. 1) to facilitate early detection of osteoporosis and provide evidence-based intervention.

## Methods

### Selection of genetic variants and data sources

To minimize the potential impact of linkage disequilibrium (LD) on the analysis, we selected SNPs that were independent of each other. Specifically, the LD of SNPs associated with Hcy was constrained to r^2^ < 0.001 within a window size of 10,000 kb, po* p* = ”1000G EUR”. As instrumental variables for the MR analysis, 18 SNPs associated with circulating serum Hcy levels at genome-wide significance (*p* < 5 × 10^–8^) in the studies of the GWAS Catalog, including 44,147 individuals of European ancestry, were utilized^14^. The corresponding summary statistics for the associations between the Hcy-associated SNPs and F-BMD from a GWAS of 8143 individuals (ID: ieu-a-997), L-BMD from a GWAS of 28,498 individuals (ID: ieu-a-982), and H-BMD from a GWAS of 142,487 individuals(ID: ieu-a-GCST006288), of European ancestry in the GEFOS consortium UK Biobank (Neale Lab) were obtained. F-BMD, L-BMD, and H-BMD were derived from dual-energy X-ray absorptiometry. Relevant ethics committees approved all studies contributing data to these analyses.

Based on the selecting principle of IVs in MR analysis, these variants were in LD, so 5 SNPs were excluded (rs7422339, rs12134663, rs957140, rs12821383, and rs2851391). Hypertension^15^, serum Vit-B_12_^15^, high uric acid^16^, and lipid metabolism disorders^17^ are likely to be important confounders in the context of the Hcy-BMD relationship. PhenoScanner (www.phenoscanner.medschl.cam.ac.uk.) was used to assess the association of these SNPs with confounders such as hypertension, serum folic acid, high uric acid, and lipid metabolism disorders. 13 of the SNPs that were significantly associated with Hcy. SNP (rs154657) significantly correlated with hypertension, SNP (rs548987) significantly correlated with uric acid, SNP (rs1801222) significantly correlated with folic acid, and 2 SNPs (rs838133 and rs2251468) significantly correlated with lipid metabolism were excluded (Table S1). Eight SNPs strongly associated with homocysteine were included as final instrumental factors (Table 1). In order to standardize the effect alleles direction in the exposure dataset, data harmonization was performed between the exposure dataset and the outcome dataset.

**Table 1:** The characteristics of the 8 SNPs associated with circulating serum homocysteine concentration.

SNPs	Chr	Pos	Nearest gene	EA	OA	EAF	beta (95% CI)	se	*P* value
rs1801133	1	11796321	MTHFR	A	G	0.34	0.158 (0.138, 0.179)	0.011	4.00e^−104^
rs2275565	1	236885376	MTR	T	G	0.21	− 0.054 (− 0.071, − 0.038)	0.009	2.00e^−10^
rs9369898	6	49414480	MUT	A	G	0.62	0.045 (0.031, 0.059)	0.007	2.00e^−10^
rs12921383	16	89859753	DPEP1/ FANCA	C	T	0.87	− 0.090 (− 0.007, − 0.063)	0.014	8.22e^−11^
rs234709	21	43066854	CBS	T	C	0.45	0.072 (0.058, 0.086)	0.007	4.00e^−24^
rs4660306	1	45513003	MMACHC	T	C	0.33	0.044 (0.030, 0.057)	0.007	2.00e^−09^
rs42648	7	90348446	GTPB10	A	G	0.40	0.0340 (0.026, 0.053)	0.007	2.00e^−08^
rs12780845	10	17181245	CUBN	A	G	0.65	0.053 (0.035, 0.071)	0.009	8.00e^−10^

*Chr* indicates chromosome, *EA* effect allele, *OA* other allele, *EAF* effect allele frequency, *se* standard error, *95% CI* 95% confidence interval.

### Statistical analyses for MR

Three MR approaches, including IVW, MR-Egger, and WM, were executed using the Two Sample MR packages in R version 4.2.2 (www.r-project.org). The results were reported as the mean effect per 1-SD genetically predicted rise in serum Hcy (log-transformed) and a 95% confidence interval (95% CI). In addition, a three-sided *p* < 0.017 = 0.05/3 was statistically significant.

### Heterogeneity and sensitivity tests

The Cochran's Q test was used to identify heterogeneity among instrumental variables derived from IVW estimation. *P* < 0.05 was deemed statistically significant, indicating that heterogeneity may occur. The MR-Egger intercept was used to identify the directional pleiotropy of instrumental variables and was generated from the MR-Egger regression. When *P* < 0.05, it was deemed statistically significant, indicating that directional pleiotropy may occur. The leave-one-out test was employed to examine the strength of instrumental factors.

### Ethics approval and consent to participate

No need for ethical approval as used of anonymous open data.

## Results

### MR analyses results

**Figure 2:**
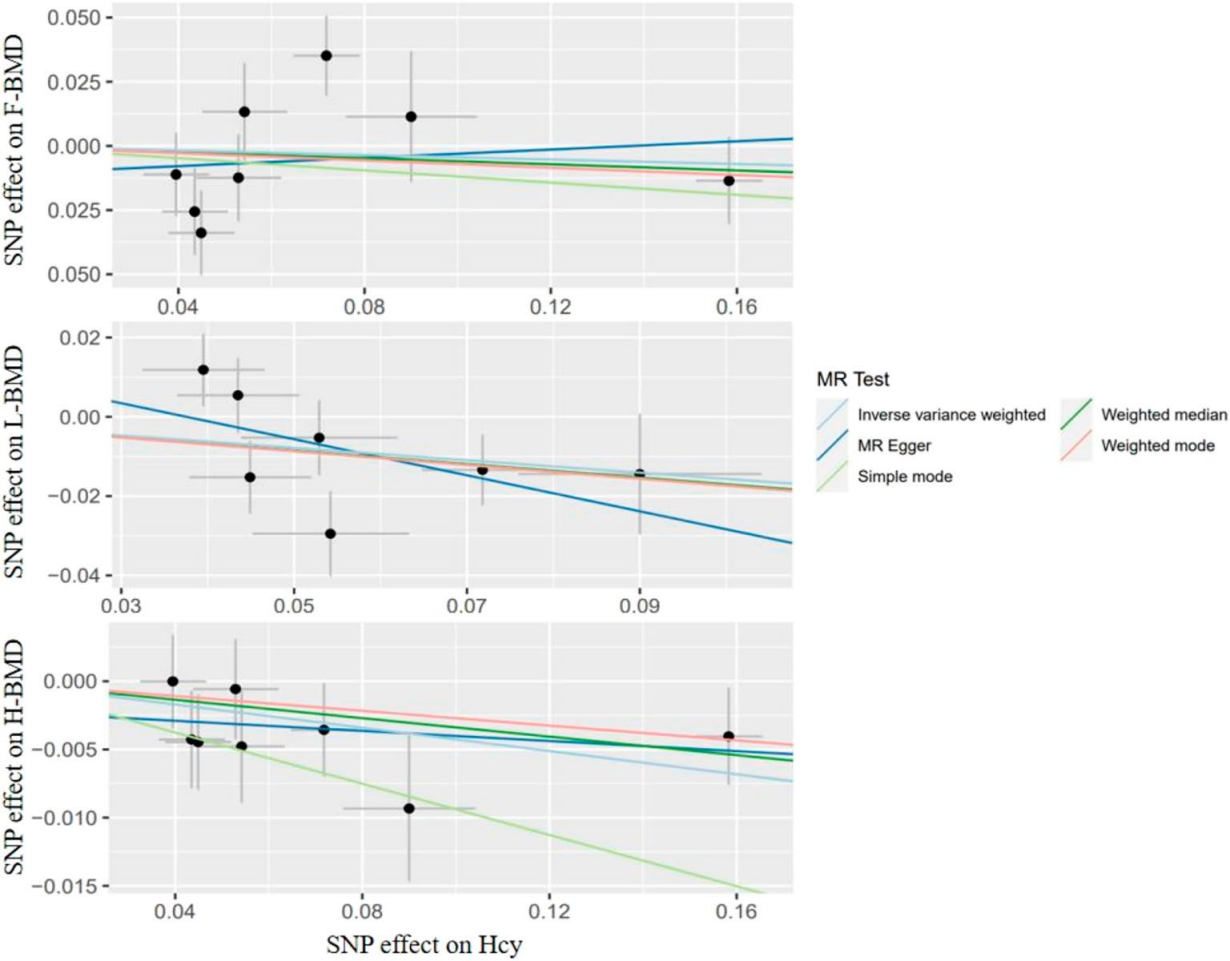
A scatter plot illustrating the causal relationship between Hcy and the risk of osteoporosis. The slope of the straight line represents the magnitude of the causal relationship. IVW, inverse-variance weighted; MR, Mendelian randomization; Hcy, homocysteine; F-BMD, forearm bone mineral density; L-BMD, lumbar bone mineral density; H-BMD, heel bone mineral density.

**Figure 3:**
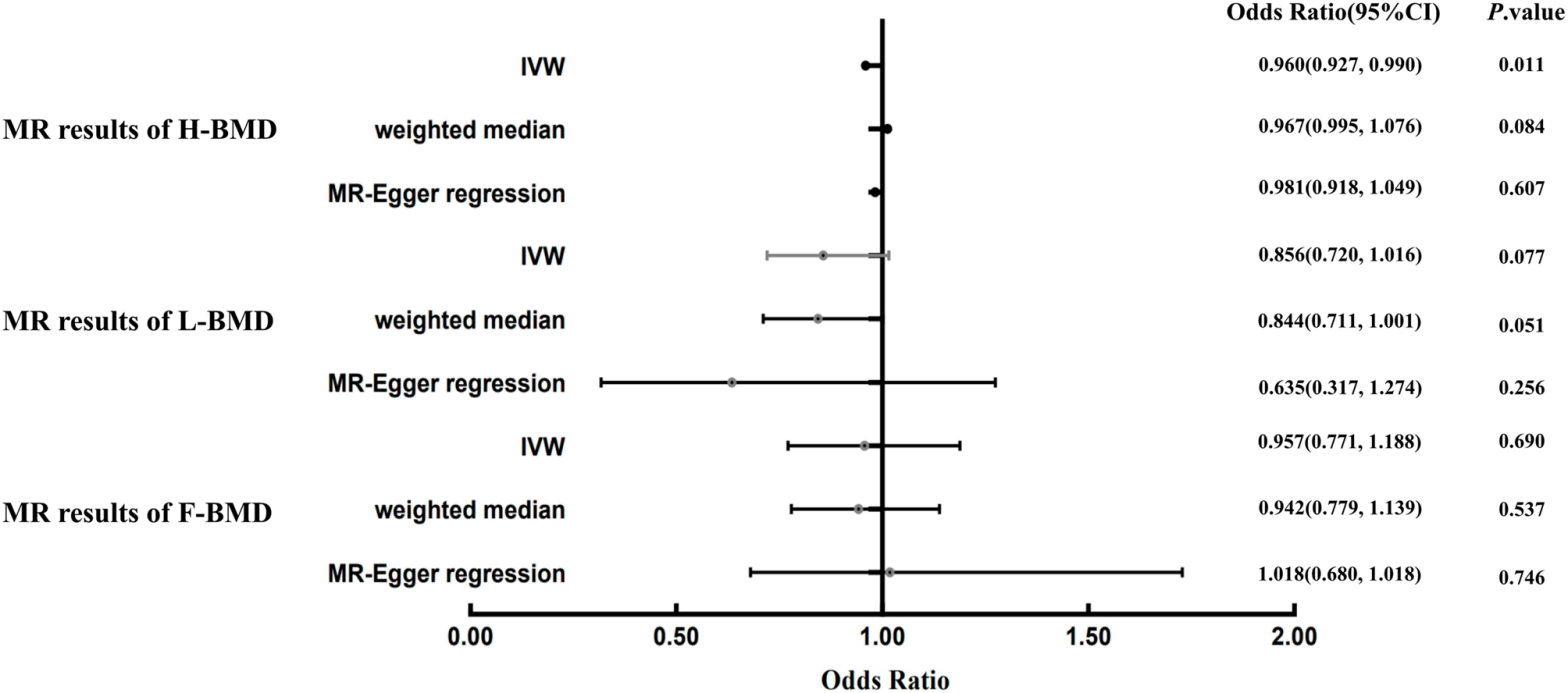
Forest plot to visualize the causal effect of Hcy on the risk of osteoporosis. IVW, inverse-variance weighted; MR, Mendelian randomization; Hcy, homocysteine; F-BMD, forearm bone mineral density; L-BMD, lumbar bone mineral density; H-BMD, heel bone mineral density.

**Figure 4:**
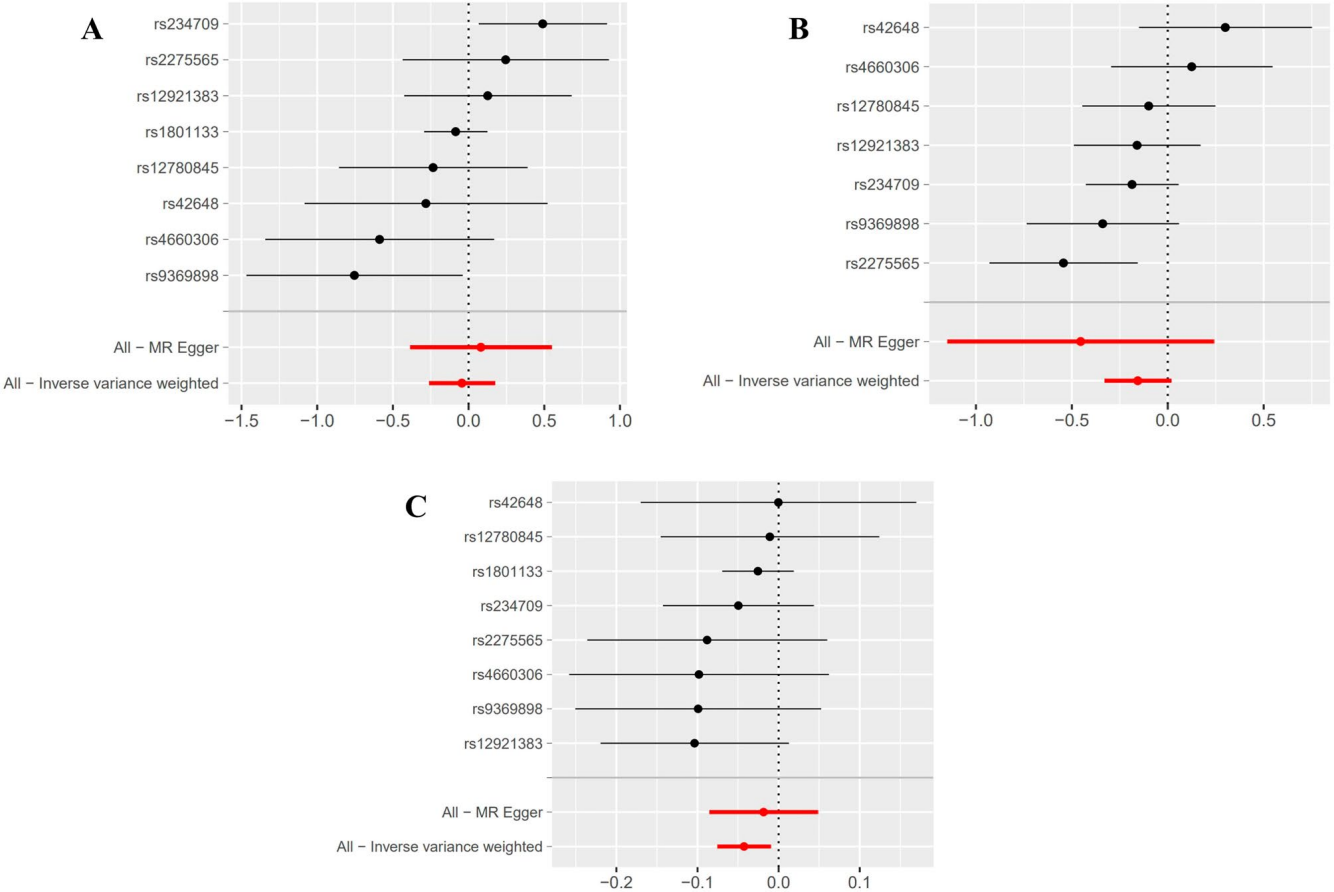
Forest plot to visualize the causality of every single SNP on osteoporosis risk. (**A**) Hcy on F-BMD risk, (**B**) Hcy on L-BMD risk, (**C**) Hcy on H-BMD risk.

Using IVW, MR-Egger, and WM regressions, the causal relationship between serum Hcy levels and osteoporosis indicators (F-BMD, L-BMD, and H-BMD) was analyzed. Upon examining the causal relationship between genetically predicted Hcy and F-BMD, and L-BMD using the IVW approach, no significant causal association was observed (all *p* value > 0.017) (Table 2). Furthermore, the regression slopes were observed to be in the same direction (Fig. 2). Conversely, utilizing the IVW technique, a causal and inverse relationship was found between genetically predicted Hcy and the H-BMD risk using the IVW technique [OR = 0.96, 95% CI = 0.927–0.990, *p* = 0.011] (Table 2). Similar trends were seen in the WM regression estimations [OR = 0.97, 95% CI = 0.995–1.076, *p* = 0.084]. Statistical significance was not found using the MR-Egger technique (OR = 0.98, 95% CI = 0.918–1.049, *p* = 0.609) (Fig. 3). Nonetheless, the consistency in the direction of the estimates generated by the three approaches lends credence to the findings' veracity. Evidently, from Figs. 2 and 4, each genetic variant contributes to the development of osteoporosis.

**Table 2:** Results of MR Analyses on the relationship of Hcy to BMDs.

Outcome	Exposure	Methods	Nsnp	beta (95% CI)	se	*P*
F-BMD	Homocysteine	MR-Egger regression	8	0.081 (− 0.385, 0.547)	0.238	0.746
		weighted median		− 0.060 (− 0.250, 0.130)	0.097	0.537
		IVW		− 0.044 (− 0.260,0.172)	0.110	0.690
L-BMD	Homocysteine	MR-Egger regression	7	− 0.454 (− 1.150, 0.242)	0.355	0.256
		weighted median		− 0.170 (− 0.341, 0.001)	0.087	0.051
		IVW		− 0.156 (− 0.328, 0.016)	0.088	0.077
H-BMD	Homocysteine	MR-Egger regression	8	− 0.019 (− 0.086, 0.048)	0.034	0.607
		weighted median		− 0.034 (− 0.005,0.073)	0.020	0.084
		IVW		− 0.043 (− 0.076, − 0.010)	0.017	0.011

*F-BMD* forearm bone mineral density, *L-BMD* lumbar bone mineral density, *H-BMD* heel bone mineral density, *MR* mendelian randomization, *95% CI* 95% confidence interval, *se* standard error, *IVW* inversevariance weighted; *P* < 0.017 is considered statistically significant.

### Heterogeneity and sensitivity analysis

**Figure 5:**
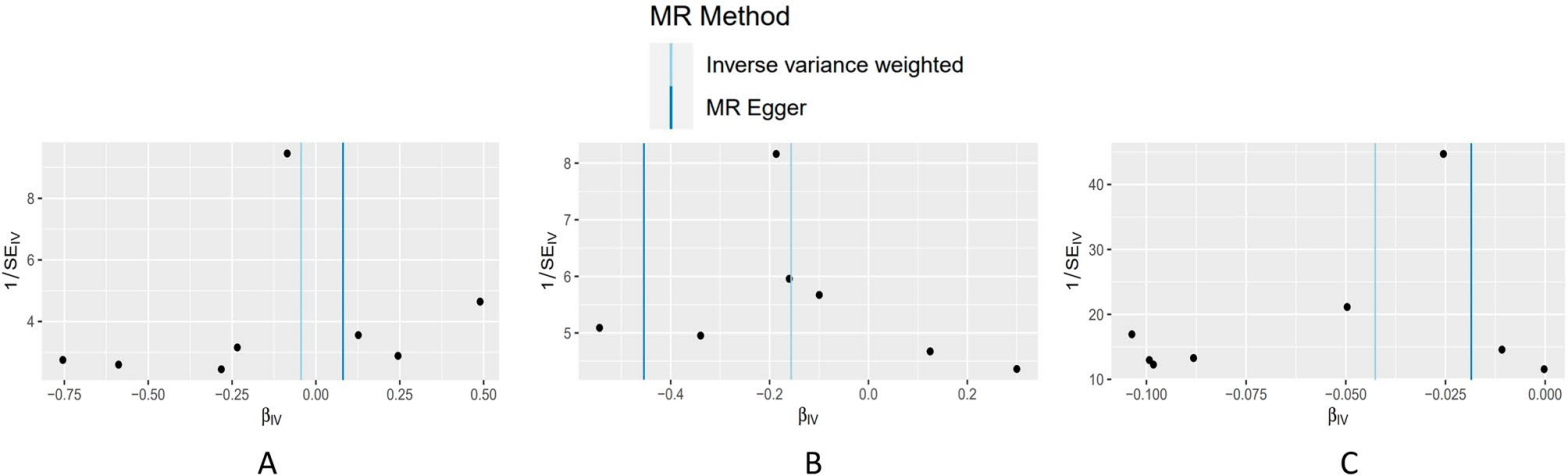
Funnel plots to visualize the overall heterogeneity of MR estimates for the effect of Hcy on osteoporosis. (**A**) Hcy on F-BMD risk, (**B**) Hcy on L-BMD risk, (**C**) Hcy on H-BMD risk; MR, Mendelian randomization; Hcy, homocysteine; F-BMD, forearm bone mineral density; L-BMD, lumbar bone mineral density; H-BMD, heel bone mineral density.

**Figure 6:**
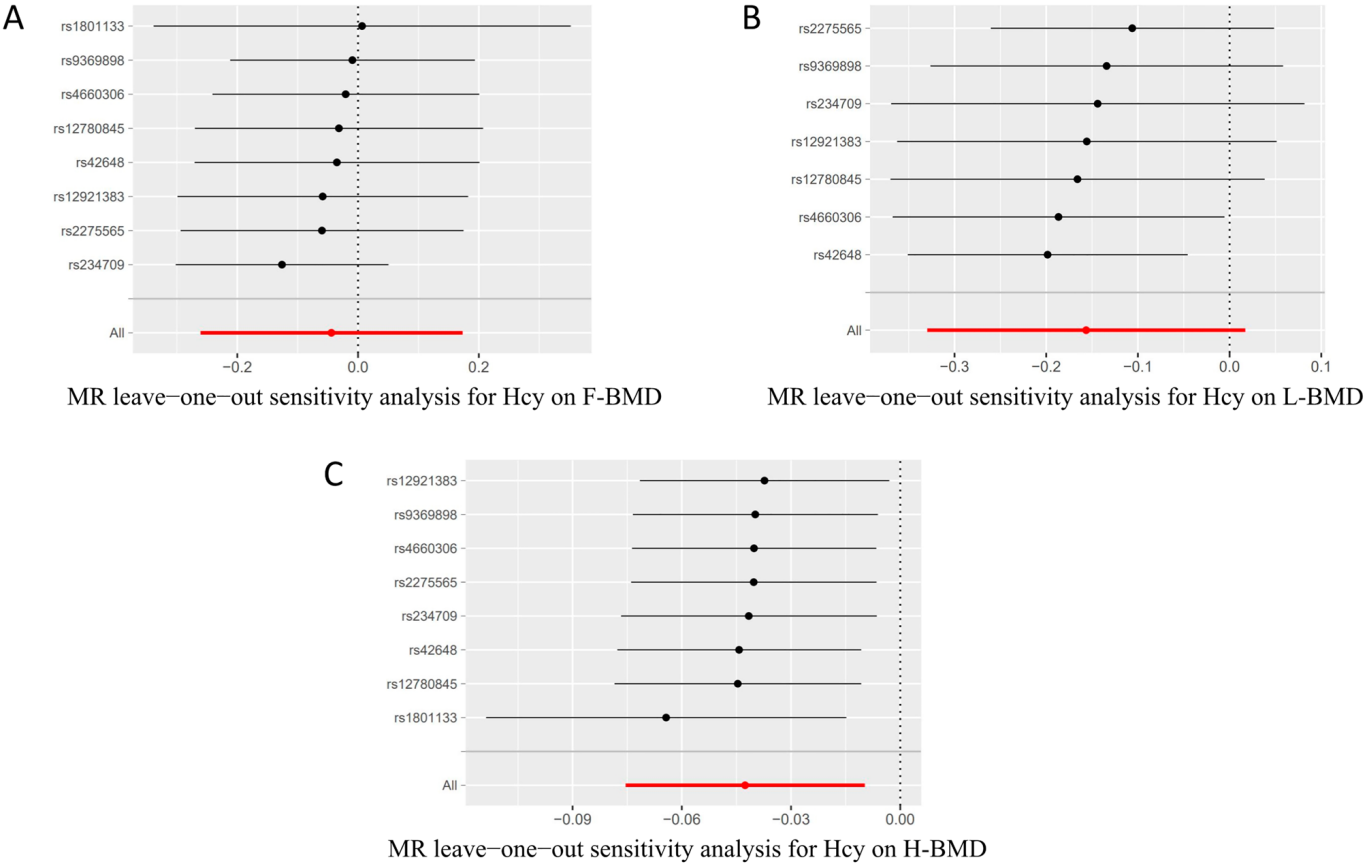
Leave-one-out plot to visualize the causal effect of Hcy on the risk of osteoporosis when leaving one SNP out. MR, Mendelian randomization; Hcy, homocysteine; F-BMD, forearm bone mineral density; L-BMD, lumbar bone mineral density; H-BMD, heel bone mineral density.

The IVW approach revealed no heterogeneity among the eight SNPs significantly associated with Hcy (*p* value > 0.017) (Table S2). The funnel plot's impact on causation was roughly symmetrical (Fig. 5). The MR-Egger-regression showed no directional pleiotropy (Hcy to F-BMD: Egger intercept = − 0.011, SE = 0.018, *p* = 0570; Hcy to L-BMD: Egger intercept = 0.017, SE = 0.120, *p* = 0.425; Hcy to H-BMD: Egger intercept = − 0.002, SE = 0.002, *p* = 0.4449). The leave-one-out analysis showed that a single SNP did not drive the MR analysis results (Fig. 6).

## Discussion

Mendelian randomization was employed for the first time in this investigation to examine the association between Hcy and OP in the European population. The findings revealed a causal relationship between serum Hcy and H-BMD, but but no causal relationship existed between Hcy and F-BMD and L-BMD.

As mentioned previously, the three assumptions of the MR Model must be satisfied for causal estimates to be accurate. Thirteen highly-associated and independent SNPs closely linked to Hcy were identified in this study, satisfying the first assumption. Furthermore, the exclusion of five SNPs (rs838133, rs154657, rs548987, rs1801222, and rs2251468) connected with confounding variables, such as lipid metabolism, hypertension, uric acid, and vitamin B, as documented in previous studies, mitigated the bias caused by a polymorphism in MR trials, satisfying the second assumption. Additionally, the MR-Egger intercepts were near 0 in this investigation (*p* > 0.05), implying that unknown variables did not lead to pleiotropy, satisfying the third assumption. Therefore, the SNPs used and the outcomes of the research are credible.

BMD is a crucial indicator for the diagnosis and evaluation of osteoporotic fractures. Studying 188 postmenopausal Moroccan women, Quzzif et al. found that plasma Hcy was significantly higher in the osteoporosis group than in the non-osteoporosis group and that Hcy was inversely linked with lumbar and total hip BMD^18^. Significant negative correlations were discovered between Hcy and femoral neck BMD (beta = 0.032, *p* = 0.010) and lumbar spine (beta = − 0.098, *p* = 0.021) in a large cross-sectional study (n = 6107). Bone mineral density was found to be affected by serum folate and body mass index but not by Hcy, as shown by a stepwise regression analysis^19,20^. Although the association between serum Hcy and BMD remains contentious, increasing Hcy levels increase the OP risk, and it is commonly acknowledged that Hcy is a risk factor for OP and osteoporotic fractures.

There are limitations in this MR. First, although the causal association between Hcy and H-BMD was verified in this investigation, it was not established between Hcy, F-BMD, and L-BMD mainly because the sample size was insufficient to provide meaningful data. Second, we used aggregated GWAS data, and the lack of specific information on sex and age prevented us from conducting subgroup analysis. Third, since the research was based on genetic information from European groups, it needs to be seen whether the findings apply to Asian people. Fourth, due to the absence of GWAS data, this research gathered only H-BMD, F-BMD, and L-BMD data. Other BMD indices, such as femoral neck bone density and hip bone density, should be included in future research to get more trustworthy findings. Finally, although we obtained a small OR, focusing on the causal relationship between Hcy and H-BMD is warranted because of the large prevalence.

In conclusion, our MR investigation revealed evidence of a causal relationship between circulating serum Hcy levels and H-BMD, but not OP. This finding needs to be further verified in a study with a larger sample size.

## Supplementary Information

Supplementary Information.

## Data Availability

The data and material that support the findings of this study are available from public datasets that could be found in GWAS Catalog.
