# Supplementary material for: Genetically predicted circulating serum homocysteine levels on osteoporosis: a two-sample mendelian randomization study
Source: Sci Rep. 2023 Jun 4;13:9063. doi: 10.1038/s41598-023-35472-2 (PMC10239750; doi:10.1038/s41598-023-35472-2)
Supplement: Supplementary file 1 — Supplementary Information. [file 41598_2023_35472_MOESM1_ESM.docx]

| **SNPs** | **Position** | **EA(OA)** | **Trait** | **beta** | ***P*** |
| --- | --- | --- | --- | --- | --- |
| rs838133 | chr19:49259529 | G(A) | total cholesterol | -0.032 | 3.65e^-10^ |
| rs154657 | chr16:89708096 | G(A) | hypertension | 0.008 | 5.14e^-13^ |
| rs548987 | chr6:25869371 | G(C) | serum urate | -0.053 | 2.51e^-09^ |
| rs1801222 | chr10:17156151 | G(A) | Vit-B levels | -0.218 | 2.00e^-13^ |
| rs2251468 | chr12:121405126 | A(C) | LDL-c | -0.032 | 2.17e^-16^ |

**Table.S1 The characteristics of the excluded SNPs aassociated with various confounding factor**

**Note:** chr, chromosome; EA, effect allele; OA, other allele;

**Table.S2 Heterogeneity test assesses whether heterogeneity exists in SNPs**

| **Outcome** | **Exposure** | **Method** | **Q statistic** | **df** | ***P*** |
| --- | --- | --- | --- | --- | --- |
| **F-BMD** | homocysteine | Inversevariance weighted | 13.880 | 7 | 0.053 |
| **L-BMD** | homocysteine | Inversevariance weighted | 10.571 | 6 | 0.103 |
| **H-BMD** | homocysteine | Inversevariance weighted | 3.500 | 7 | 0.835 |

**Note:** p<0.05 is considered statistically significant; F-BMD, forearm bone mineral density; L-BMD, lumbar bone mineral density; H-BMD, heel bone mineral density; df, degree of freedom.
